# Resolving heterogeneity in first-episode and drug-naive major depressive disorder based on individualized structural covariance network: evidence from the REST-meta-MDD consortium

**DOI:** 10.1017/S0033291725100664

**Published:** 2025-06-24

**Authors:** Songhao Hu, Li Zhu, Xiangyang Zhang

**Affiliations:** 1 Fourth People’s Hospital in Hefei, Affiliated Psychological Hospital of Anhui Medical University, Hefei, China; 2 School of Mental Health and Psychological Sciences, Anhui Medical University, Hefei, China

**Keywords:** major depressive disorder, heterogeneity, structural covariance network

## Abstract

**Background:**

Major depressive disorder (MDD) is a complex and heterogeneous disorder, and this heterogeneity poses a significant challenge for advancing precision medicine in patients with MDD. MRI-based subtyping analysis has been widely employed to address the heterogeneity of MDD patients. In this study, we investigated the subtypes of first-episode and drug-naive (FEDN) MDD patients based on the individualized structural covariance network (IDSCN).

**Methods:**

In this study, we used T1-weighted anatomical images of 164 FEDN MDD patients and 164 healthy controls from the REST-meta-MDD consortium. The IDSCN of participants was obtained using the network template perturbation method. Subtypes of FEDN MDD were identified using k-means clustering analysis, and differences in neuroimaging findings and clinical symptoms between the identified subtypes were compared using two-sample *t*-tests.

**Results:**

This study identified two subtypes of FEDN MDD: subtype 1 (*n* = 117) and subtype 2 (*n* = 47) by characterizing 10 edges that were significantly altered in at least 5% of patients (i.e., 8 patients) in the IDSCN. Compared with subtype 2, subtype 1 had significantly higher anxiety symptom scores, stronger structural covariance edges in 9 edges within the thalamus, and a significantly reduced gray matter volume (GMV) in the frontal and parietal regions, and in the thalamus.

**Conclusions:**

Our results suggest that patients with FEDN MDD can be classified into two different subtypes based on their IDSCN, providing an important reference for personalized treatment and precision medicine for patients with FEDN MDD.

## Introduction

Depression is a prevalent mood disorder that imposes a significant burden on the world, both through direct medical costs and indirect productivity losses (Gore et al., 2011; McCarron, Shapiro, Rawles, & Luo, 2021). A large number of patients with depression do not receive timely medication, which may contribute to the development of major depressive disorder (MDD) (M. Zhang, 2010; Zhu et al., 2023). The lifetime prevalence of MDD, the second leading cause of disability in the Chinese population, is approximately 1.8% (G. Yang et al., 2013). A cross-sectional study conducted in China revealed that only 0.5% of patients diagnosed with MDD received medication or psychotherapy (Lu et al., 2021). This considerable discrepancy between the high prevalence of the disorder and the low treatment rate makes MDD a major public health problem that requires urgent attention in the realm of mental health in China. Unfortunately, our understanding of MDD remains limited, especially given the highly heterogeneous nature of the disorder, which is characterized by complex clinical subtypes and diverse symptoms (Lynch, Gunning, & Liston, 2020).

Currently, the pathogenesis of MDD remains elusive, leading to significant heterogeneity across various domains such as symptomatology, biochemistry, genetics, and neuroimaging (Huang, Li, Shen, Liang, & Li, 2023; Kunugi, Hori, & Ogawa, 2015; B. Zhang et al., 2023; Drysdale et al., 2017). To address this heterogeneity, classifying MDD into different subtypes is a promising approach (Buch & Liston, 2021; Grosenick et al., 2019; Han, Xu et al., 2023). Researchers have categorized MDD from different perspectives (Huang et al., 2023; Martin et al., 2020; Meng et al., 2022). For example, Martin et al. (2020) aimed to classify patients with MDD based on cognitive symptoms, whereas Huang et al. (2023) identified two different subtypes of MDD based on the expression of endoplasmic reticulum stress genes. However, these classification methods are often based on phenomenological or genetic criteria only, resulting in a weak link to the underlying neuroanatomical pathophysiology. Phenomenology-based classification methods fail to establish clear diagnostic thresholds, while classification methods based on genetic factors have limited clinical relevance (Han, Xu, et al., 2023; Okada et al., 2015). Therefore, there is a need to investigate alternative classification methods to better understand the heterogeneity of MDD.

In recent years, magnetic resonance imaging (MRI) has become a widely used tool for studying MDD disease-related patterns (Rolls et al., 2018; F.-F. Zhang, Peng, Sweeney, Jia, & Gong, 2018). Brain structure covariance-based networks represent a cutting-edge concept in this field. It suggests that inter-individual variations in brain morphological features tend to show covariation with changes in other brain regions (Alexander-Bloch, Giedd, & Bullmore, 2013; Chong, Dumkrieger, & Schwedt, 2017). This phenomenon reflects the underlying functional organization of the network and highlights anatomical connectivity (Chong et al., 2017; Lerch et al., 2006). In a previous study, Xiong et al. (2021) reported that patients with current MDD and those with a history of MDD exhibited reduced path length and small-world properties in their structural covariance networks. In addition, Nestor et al. (2022) demonstrated that these alterations in the structural covariance networks of patients with MDD were associated with their response to repetitive transcranial magnetic stimulation (rTMS) therapy. However, most of these studies have focused primarily on group-level differences, thereby neglecting heterogeneity among individuals with MDD (Wolfers et al., 2018). This emphasis on group averages may mask the unique experiences and symptoms of individual patients. Therefore, it is imperative to investigate imaging heterogeneity in MDD patients from an individual perspective.

Liu et al. proposed the network template perturbation method to calculate the individualized differential structural covariance network (IDSCN) in 2021 (Liu, Palaniyappan et al., 2021). In their study, the researchers used the IDSCN of schizophrenia patients as a feature and successfully classified these patients into two subtypes, demonstrating the heterogeneity of schizophrenia from an individual perspective. To the best of our knowledge, there is no study using the network template perturbation method to investigate the heterogeneity of first-episode and drug-naive (FEDN) MDD patients. In this study, we calculated the IDSCN of each FEDN MDD patient, classified all patients into two subtypes based on the IDSCN features, and described the associated symptomatological and neuroimaging features of each subtype in a large multicenter sample in China.

## Materials and methods

### Participants

Structural magnetic resonance imaging (sMRI) data utilized in this study were obtained from the REST-meta-MDD project consortium (Yan, Wang, Zuo, & Zang, 2016). This consortium consists of 25 research teams from 18 hospitals in China (P. Liu, Li, et al., 2021; Yan et al., 2016). Participants included in the study were required to meet the diagnostic criteria for MDD as defined by either ICD-10 or DSM-IV. For participants included in the study, the consortium members provided only basic information, including diagnosis, gender, age, educational background, and scores on the 17-item Hamilton Depression Rating Scale (HAMD-17) and the 14-item Hamilton Anxiety Rating Scale (HAMA-14). Prior to participation in the study, each participant submitted written informed consent to the local institution, which ensured the ethical integrity of the study and protected the rights of the participants.

Specific inclusion criteria for FEDN MDD patients were as follows: (1) meeting diagnostic criteria for MDD as assessed by the Structured Clinical Interview for the Diagnostic and Statistical Manual of Mental Disorders-IV (DSM-IV) or the International Classification of Diseases 10 (ICD-10); (2) a first episode of MDD; and (3) not having received any previous medication.

Exclusion criteria for all participants were as follows: (1) patients with remission or late-onset depression; (2) patients lacking basic demographic or clinical information, including gender, age, educational background, HAMD-17 scores, or HAMA-14 scores; and (3) patients with poor-quality imaging data, such as inadequate spatial normalization (Yan et al., 2019).

Based on the above criteria, we excluded 88 patients with a diagnosis of MDD and 38 healthy controls from the analysis (Yan et al., 2019). Of the eligible patients, we assessed 164 patients with FEDN MDD who met the criteria and selected 164 healthy controls (HCs) with similar sex ratios, age, and educational backgrounds to the patient group for analysis.

### Clinical measures

In this study, we used the HAMD-17 to assess the severity of depression, and the HAMA-14 to assess the severity of anxiety in FEDN MDD patients. Higher scores on these scales indicate greater severity of depression or anxiety, respectively. Both the HAMD-17 and the HAMA-14 have demonstrated good reliability and validity in the Chinese population(Maier, Buller, Philipp, & Heuser, 1988; Zheng et al., 1988).

### MRI data acquisition, and preprocessing

T1-weighted MRI data were preprocessed using DPARSF software (Chao-Gan & Yu-Feng, 2010) and then further processed using SPM 8 and VBM 8 toolbox (http://dbm.neuro.unijena.de/vb) (Yan et al., 2016). Images were normalized to template space and segmented into grey matter (GM), white matter (WM), and cerebrospinal fluid (CSF). Alignment to MNI space was done by the normalization function of the Diffeomorphic Anatomical Registration Through Exponentiated Lie algebra (DARTEL) toolbox.

Finally, the GM maps of each subject were smoothed using an 8 mm full-width at half maximum (FWHM) Gaussian kernel. In addition, we used the automated anatomical labeling atlas (AAL3) atlas (Rolls, Huang, Lin, Feng, & Joliot, 2020) to divide the brain into 166 distinct regions and extracted the grey matter volume (GMV) of these regions for each individual for subsequent analysis.

### Construction of IDSCN

**Figure 1.**
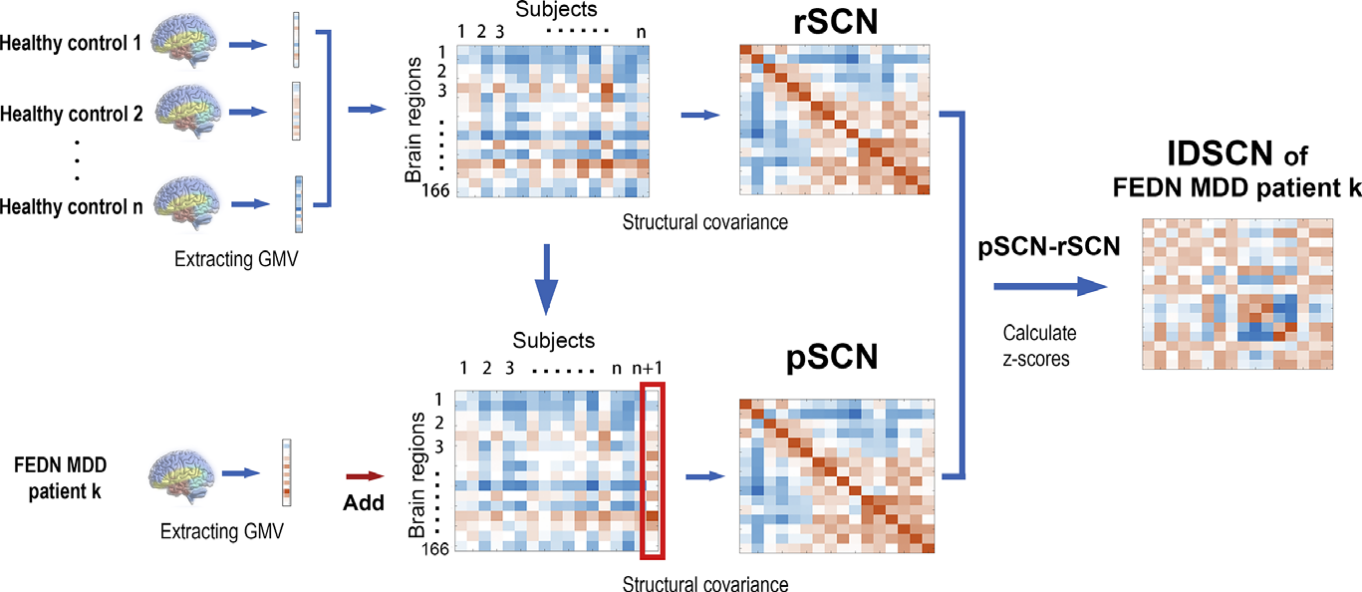
Flowchart of IDSCN construction. IDSCN, individualized structural covariance network; rSCN, reference SCN; pSCN, perturbed SCN.

We constructed the IDSCN using the network template perturbation method, and we briefly described the method here (see Figure 1; Z. Liu, Li, et al., 2021). (1) First, for the 164 HCs, we constructed the reference SCN (rSCN) by computing Pearson correlation coefficients of GMV between each pair of brain regions, with age, gender, educational level, and site information included as covariates. (2) Next, we divided a FEDN MDD patient (denoted as k) into HCs and repeated the previous step to construct the perturbed SCN (pSCN). (3) Then, we calculated the difference between the pSCN and rSCN to derive the difference SCN (dSCN), defined as dSCN = pSCN – rSCN. (4) Finally, the following equation was applied to derive the *Z*-value of dSCN to construct the IDSCN for patient *k.*

(1)

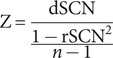

We repeated the above steps for each FEDN MDD patient, constructing an IDSCN for each individual.

### Subtyping FEDN MDD patients using the IDSCN

We calculated the *p*-value for each edge based on the *Z*-score of the edge in the IDSCN of each patient. After Bonferroni’s test (*p* < 0.001), we identified the edges in each patient’s IDSCN that were significantly different from the rSCN and counted the number of patients with significant differences in each edge. Ten edges with significant changes in at least 5% (i.e., 8 patients) of the patients were selected as features for k-mean cluster analysis. The number of clusters was set between 2 and 10, with the optimal number of subgroups determined by the maximum mean silhouette value, and 100 clusters were performed at each number of clusters to obtain the final clustering index (with maximum mean silhouette value).

### Statistical analysis

Demographic and symptom scale data of all patients were analyzed using SPSS version 23.0. The chi-square test was used for gender differences between MDD patients and HCs and between the two subtypes of FEDN MDD patients. Two-sample *t*-tests were performed for continuous variables such as age, education, and symptom scores. The significance threshold for differences in data and clinical symptom scores was set at a *p-*value of <0.05.

To compare IDSCN edges between subtypes, we used the GRETNA toolkit with gender, age, education, and site information as covariates and analyzed the data using a two-sample *t*-test (Bonferroni corrected *p* < 0.05). In addition, we performed Pearson correlation analyses to investigate the relationship between altered edges and clinical symptom scores.

We used a two-sample *t*-test in the SPM12 software (http://www.fil.ion.ucl.ac.uk/spm/software/spm12/) to compare GMV in the two subtypes of patients with FEDN MDD. In addition, we included gender, age, education, and site information as covariates in the analyses. Multiple comparisons were controlled with a cluster-extent threshold combined with a height threshold of 0.001, to produce clusters with a family-wise error (FWE) of *p* < 0.05.

## Results

### Demographics

After quality control, a total of 164 patients with MDD and 164 HCs were included in this study. The demographic data and clinical characteristics of all subjects are shown in Table 1. There were no significant differences between MDD patients and HCs in terms of gender, age, and education (*p* > 0.05).

**Table 1.** Demographic and clinical characteristics of all participants

Characteristics (mean ± SD)	MDD (*n* = 164)	HC (*n* = 164)	Statistical value	*p*-value
Gender (M/F)^a^	57/107	69/95	1.856	0.173
Age (years)^b^	35.02 ± 13.09	33.73 ± 14.73	0.840	0.401
Education (years)^b^	10.91 ± 3.58	10.57 ± 6.00	0.625	0.532
HAMD–17^b^	22.61 ± 5.65	NA		
HAMA–14^b^	22.60 ± 8.67	NA		

Abbreviations: HC, ‘healthy controls’; MDD, ‘major depressive disorder’.

a Chi-square test.

b Two-sample *t* tests.

* *p* < 0.05.

### Heterogeneity of IDSCNs in FEDN MDD patients

We constructed an IDSCN for each FEDN MDD patient based on AAL3. Subsequently, we obtained differential structural covariance edges for each FEDN MDD patient (*p* < 0.001, Bonferroni corrected 13695 edges). Of these difference edges, 10641 were shared by at least one patient, whereas 2635 difference edges were shared by at least two patients, further demonstrating the heterogeneity of covariance edges in the FEDN MDD structure.

### Subtypes of FEDN MDD patients identified by IDSCN

We sorted all edges in descending order based on the number of patients exhibiting significant edge variation. We used the *Z*-scores of the top 10 connected edges that exhibited significant differences in at least 5% of patients as features for k-means clustering. When the silhouette coefficient reached its maximum value, we determined that the optimal number of clusters was 2. Therefore, we divided the FEDN MDD patients into two subgroups, which were identified as two different subtypes of FEDN MDD patients (subtype 1, *n* = 117; subtype 2, *n* = 47).

**Figure 2.**
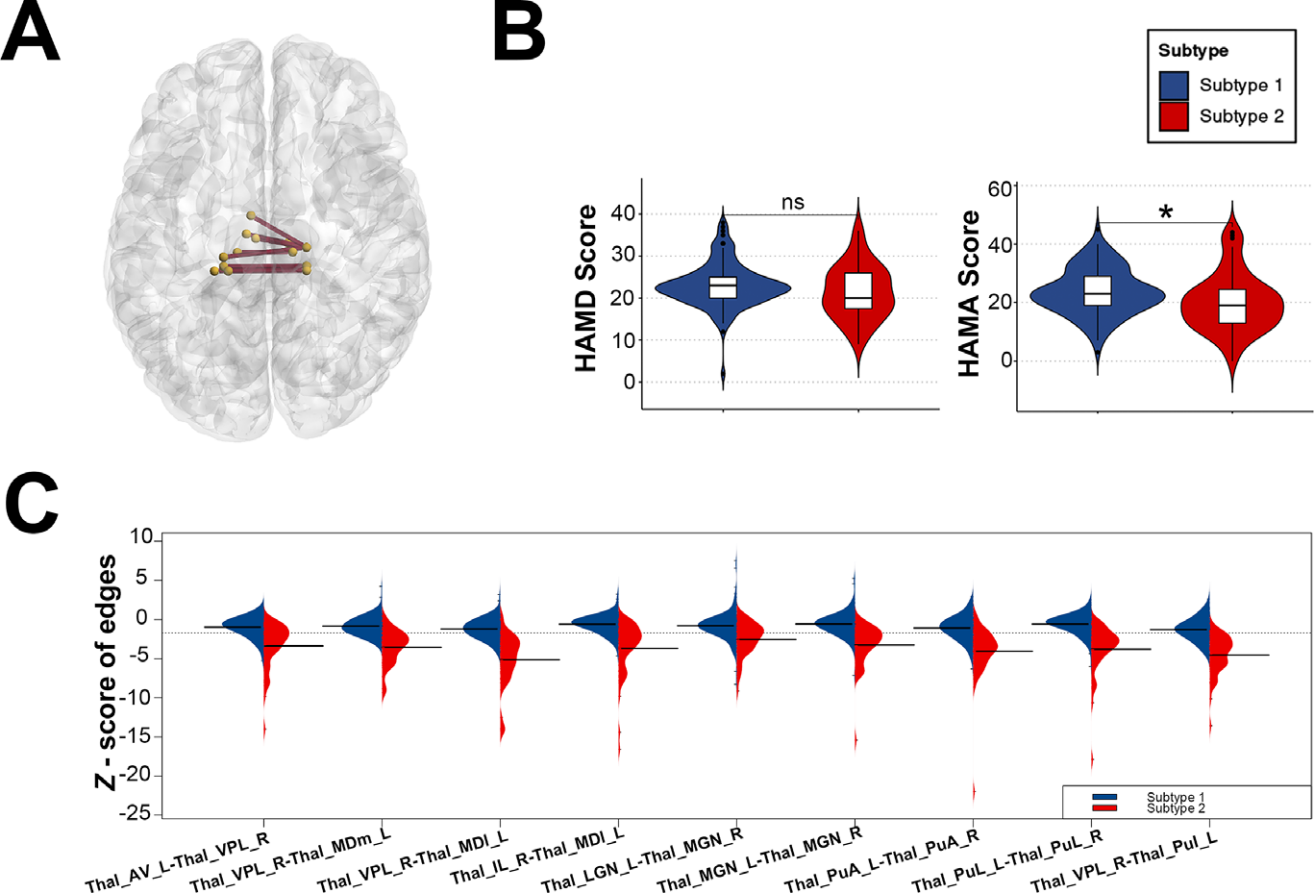
(A) Structural covariance edges significantly stronger in subtype 1 than in subtype 2; (B) Differences in symptom scale scores between the two subtypes; (C) Differences in *Z*-scores of structural covariate edges between the two subtypes. Thal_AV_L = Thalamus, Anteroventral, Left;Thal_VPL_R = Thalamus, Ventral posterolateral, Right; Thal_MDm_L = Thalamus, Mediodorsal medial magnocellular, Left;Thal_IL_R = Thalamus, Intralaminar, Right; Thal_MDl_L = Thalamus, Mediodorsal lateral parvocellular, Left;Thal_LGN_L = Thalamus, Lateral geniculate, Left;Thal_MGN_R = Thalamus, Medial Geniculate, Right;Thal_MGN_L = Thalamus, Medial Geniculate, Left;Thal_PuA_L = Thalamus, Pulvinar anterior, Left;Thal_PuA_R = Thalamus, Pulvinar anterior,Right;Thal_PuL_L = Thalamus, Pulvinar lateral, Left;Thal_PuL_R = Thalamus,Pulvinar lateral, Right;Thal_PuI_L = Thalamus, Pulvinar inferior, Left.

In addition, we compared the general demographic data of the two FEDN MDD subtypes and found no differences between the two subtypes in terms of age distribution and gender; however, there was a significant difference in terms of education (*t* = 2.230, *p* = 0.027) (see Table 2). In terms of clinical symptoms, patients with both FEDN MDD subtypes had similar levels of depressive symptoms; however, patients with subtype 2 had significantly higher levels of anxiety symptoms compared to subtype 1 (*t* = 2.412, *p* = 0.017) (see Table 2 and Figure 2B).

**Table 2.** Demographic and clinical characteristics of two FEDN MDD subtypes

Characteristics (mean ± SD)	Subtype 1 (*n* = 117)	Subtype 2 (*n* = 47)	Statistical value	*p*-value
Gender (M/F)^a^	38/79	19/28	0.934	0.334
Age (years)^b^	35.91 ± 12.84	32.79 ± 13.57	1.388	0.167
Education (years)^b^	11.30 ± 3.56	9.94 ± 3.50	2.230	0.027*
HAMD–17^b^	23.07 ± 5.30	21.47 ± 6.36	1.649	0.101
HAMA–14^b^	23.62 ± 7.92	20.06 ± 9.95	2.412	0.017*

Abbreviations: HC, ‘healthy controls’; MDD, ‘major depressive disorder’.

a Chi-square test.

b Two-sample *t*-tests.

* *p* < 0.05.

### Differential structural covariance edges for the two FEDN MDD subtypes

**Figure 3.**
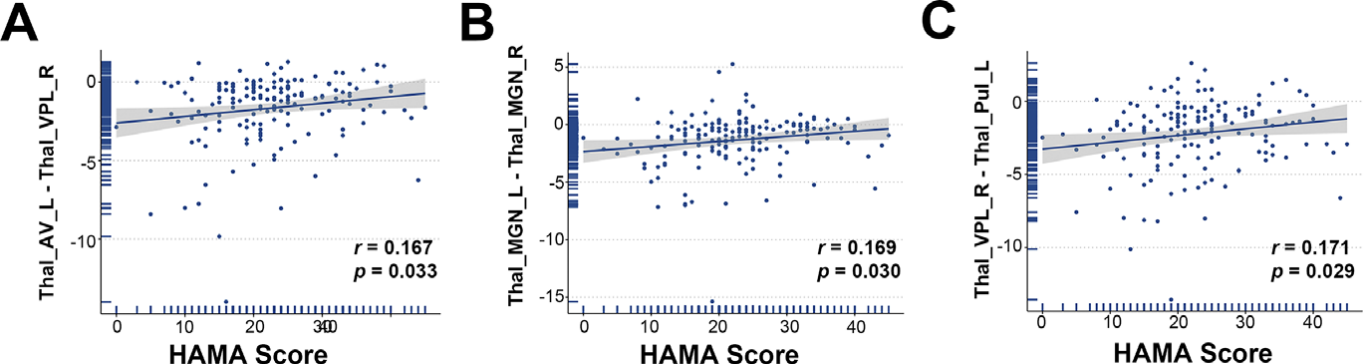
Scatterplot of the correlation between *Z*-scores of structural covariance edges and HAMA scores. Thal_AV_L = Thalamus, Anteroventral, Left; Thal_VPL_R = Thalamus, Ventral posterolateral, Right;Thal_MGN_R = Thalamus, Medial Geniculate, Right;Thal_MGN_L = Thalamus, Medial Geniculate, Left;Thal_PuI_L = Thalamus, Pulvinar inferior, Left.

Nine of the top 10 structural covariance edges of the IDSCN were significantly different between the two FEDN MDD subtypes (Bonferroni corrected *p* < 0.05; Figure 2A). All nine edges belonged to the same category, and the *Z*-scores of their connecting edges were significantly higher in subtype 1 than in subtype 2 (Figure 2C and Table 3). These edges were mainly located in the thalamus. As shown in Figure 3, the *Z*-scores for all three edges, from the left anteroventral region of the thalamus to the right Ventral posterolateral region of the thalamus (Thal_AV_L-Thal_VPL_R), from the left medial geniculate region of the thalamus to the right medial geniculate region of the thalamus (Thal_MGN_L-Thal_MGN_R), and from the right ventral posterolateral region of the thalamus to the left pulvinar inferior region of the thalamus (Thal_VPL_R-Thal_PuI_L), were positively correlated with the anxiety symptom scores.

**Table 3.** Structural covariance edges significantly stronger in subtype 1 than in subtype 2

Seed region	Target region	*Z*-scores of edges	*t*-value	*p*-value
Thal_AV_L	Thal_VPL_R	−1.66 ± 2.18	6.283	<0.001
Thal_VPL_R	Thal_MDm_L	−1.62 ± 1.97	8.657	<0.001
Thal_VPL_R	Thal_MDl_L	−2.33 ± 2.77	9.268	<0.001
Thal_IL_R	Thal_MDl_L	−1.49 ± 2.53	7.618	<0.001
Thal_LGN_L	Thal_MGN_R	−1.29 ± 2.17	5.778	<0.001
Thal_MGN_L	Thal_MGN_R	−1.35 ± 2.26	8.516	<0.001
Thal_PuA_L	Thal_PuA_R	−1.94 ± 2.62	7.214	<0.001
Thal_PuL_L	Thal_PuL_R	−1.50 ± 2.46	9.239	<0.001
Thal_VPL_R	Thal_PuI_L	−2.22 ± 2.35	9.931	<0.001

Thal_AV_L = Thalamus, Anteroventral, Left; Thal_VPL_R = Thalamus, Ventral posterolateral, Right; Thal_MDm_L = Thalamus, Mediodorsal medial magnocellular, Left; Thal_IL_R = Thalamus, Intralaminar, Right; Thal_MDl_L = Thalamus, Mediodorsal lateral parvocellular, Left; Thal_LGN_L = Thalamus; Lateral geniculate, Left; Thal_MGN_R = Thalamus, Medial Geniculate, Right; Thal_MGN_L = Thalamus, Medial Geniculate, Left; Thal_PuA_L = Thalamus, Pulvinar anterior, Left; Thal_PuA_R = Thalamus, Pulvinar anterior, Right; Thal_PuL_L = Thalamus, Pulvinar lateral, Left; Thal_PuL_R = Thalamus, Pulvinar lateral, Right;Thal_PuI_L = Thalamus, Pulvinar inferior, Left.

### GMV differences between the two FEDN MDD subtypes

**Figure 4.**
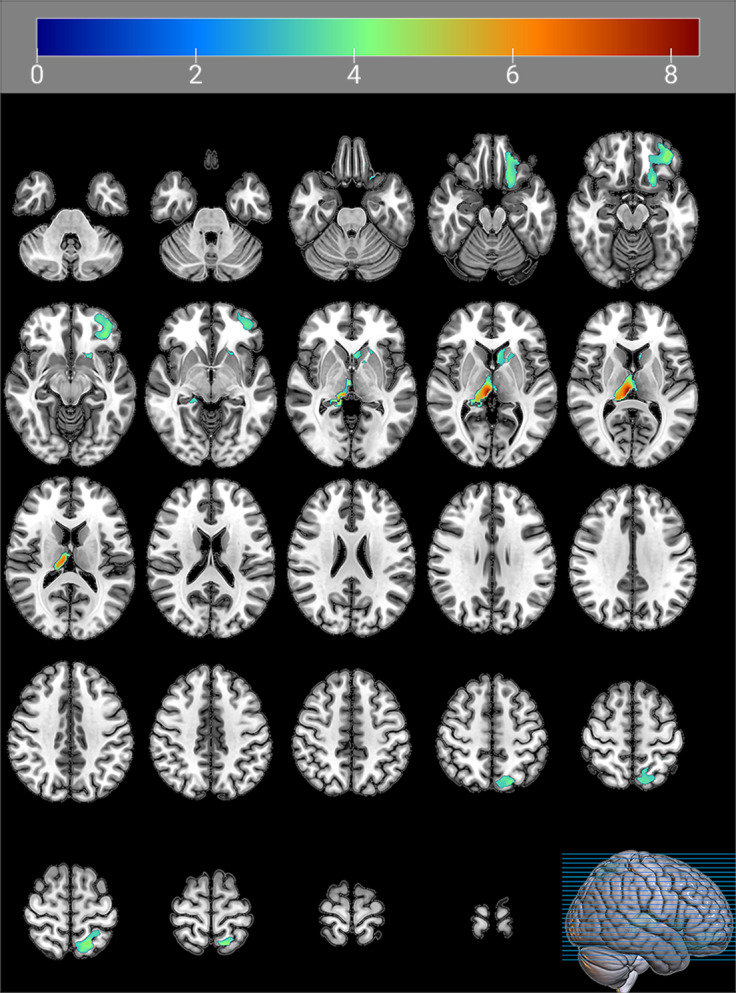
Brain regions with significantly higher GMV in subtype 2 than GMV in subtype 1. The color bars indicate the *t*-value (FWE correction, cluster-*p* < 0.05, voxel-*p* < 0.001).

Significant differences in GMV were observed in three brain regions: the left hemisphere of the inferior orbital gyrus (Frontal_Inf_Orb_2_L), the right side of the middle nucleus of the thalamic pituitary nucleus (Thal_PuM_R), and the left hemisphere of the superior parietal gyrus (Parietal_Sup_L). Subtype 1 had lower GMV in all three regions than Subtype 2 (Table 4 and Figure 4).

**Table 4.** Brain regions with significantly higher GMV in subtype 2 than GMV in subtype 1

Brain areas	Cluster size (voxels)	MNI coordinates			*t-*value (peak)
		X	Y	Z	
Frontal_Inf_Orb_2_L	2660	−39	45	−16.5	4.637
Thal_PuM_R	1405	4.5	−24	9	8.468
Parietal_Sup_L	1168	−18	−60	67.5	4.889

Abbreviations: MNI coordinates, Coordinates of primary peak locations in the Montreal Neurological Institute space; Frontal_Inf_Orb_2_L, Inferior frontal gyrus pars orbitalis, Left; Thal_PuM_R, Thalamus, Medial Geniculate, Right; Parietal_Sup_L, Superior parietal gyrus, Left.

## Discussion

In this study, we investigated the heterogeneity of FEDN MDD by IDSCN for the first time in a large multicenter sample in China. We successfully identified two distinct subtypes of FEDN MDD patients using edges in the IDSCN as features. These two subtypes differed significantly in clinical symptoms and neuroimaging findings. This study deepens our understanding of the heterogeneity of FEDN MDD patients from a neuroimaging perspective, providing new insights and informing a reference for precision medicine for FEDN MDD patients.

**Figure 5.**
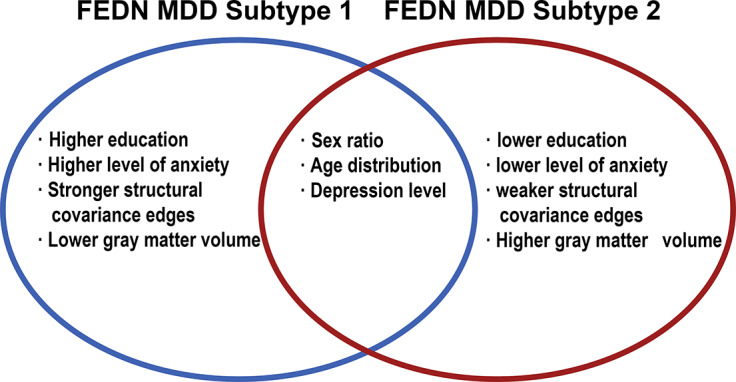
Summary of FEDN MDD subtypes.

MDD is a disorder characterized by complexity and heterogeneity (Suseelan & Pinna, 2023). A complex interplay of psychological, social, biological, and other factors contributes to the onset and progression of MDD (Albert & Newhouse, 2019; Demyttenaere et al., 2004; Ménard, Hodes, & Russo, 2016; Suseelan & Pinna, 2023). However, in clinical practice, MDD is often viewed as a universal disease, thus neglecting the various subtypes with varying manifestations. This oversight can lead to misdiagnosis of patients with MDD, adversely affecting subsequent accurate treatment (Kessler et al., 2017; Suseelan & Pinna, 2023). Misdiagnosis of MDD is very common, especially in differentiating it from bipolar disorder (Chen et al., 2019; R. Yang et al., 2023; H. Liu et al., 2018). A community-based study showed that only 38.4% of people with MDD met the diagnostic criteria (Mojtabai, 2013). Taken together, the heterogeneity of MDD poses a significant challenge to accurate diagnosis and treatment, while obscuring the underlying neuropathological mechanisms of the disease (Sun et al., 2021). In response to the heterogeneity of MDD, researchers have attempted to classify MDD into different subtypes with a biological basis. In this study, we classified patients with FEDN MDD into two subtypes (subtype 1 and subtype 2) based on their IDSCN characteristics. Notably, there were significant differences in clinical symptoms between the two FEDN MDD subtypes; specifically, subtype 1 showed more severe anxiety symptoms than subtype 2 (see Figure 5). This finding suggests that the two FEDN MDD subtypes defined by neuroimaging features can manifest distinct clinical and behavioral phenotypes.

In a previous study distinguishing subtypes based on MRI, Han, Zheng et al. (2023) used the same IDSCN approach as we did. Although Han et al. succeeded in classifying depressed patients into two strong neuroanatomical subtypes, their findings suggest that these subtypes are based purely on neuroimaging. They found differences in the IDSCN in motor and subcortical-cerebellar networks but did not observe significant differences in clinical symptoms, which contrasts with the findings in our current study. Notably, our sample was derived from the large, multi-center FEDN MDD cohort. Our study had a larger sample size, greater severity of depression, and greater representativeness than the study by Han, Zheng et al. (2023). The discrepancies between our findings and those of Han, Zheng et al. (2023) underscore that although MDD is a subtype of depression, it exhibits no less heterogeneity in neuroimaging than depression. The diagnostic criteria for MDD are more stringent than those for depression. According to the DSM-5 criteria, a diagnosis of MDD requires the presence of five out of nine symptoms, including depressed mood or anhedonia. This implies that even with such stringent criteria, there are 227 possible combinations of symptoms that can lead to an MDD diagnosis, highlighting the substantial heterogeneity of MDD (American Psychiatric Association, 2013). Therefore, identifying biologically relevant MDD diagnostic subtypes, elucidating its heterogeneity, and developing a dimensional rating system that goes beyond traditional MDD diagnostic categories are important directions for future neuroimaging research in MDD patients (Lynch et al., 2020).In addition, Yang et al. (2025) recently classified 65 patients with FEDN MDD into two neurobiological subtypes using the Louvain community detection algorithm and cortical surface area as a feature. The clustering results of the study by Yang et al. showed that morphological heterogeneity was associated with the severity of anxiety symptoms, which is in line with the results of our study, and further supports the idea that the presence of subtypes defined by morphological features is associated with anxiety symptoms.

In this study, we found significant differences in IDSCN between the two FEDN MDD subtypes. Specifically, of the 10 structural covariance edges that were altered in 5% of patients, nine edges were significantly stronger in subtype 1 than in subtype 2, all of which were located in the thalamus. As the central hub of the cerebral cortical network, the thalamus is critical to the functioning of the cerebral cortex (W. Liu et al., 2025). The thalamus is a key node for transmitting sensory signals to the brain and plays an important role in emotion regulation due to its close connections with regions such as the limbic system (Barson, Mack, & Gao, 2020; B.-Z. Li et al., 2021; Saalmann, Pinsk, Wang, Li, & Kastner, 2012). A study investigating thalamic networks showed significantly increased connectivity variability between higher-order cortical networks and the thalamus in patients with MDD compared to healthy individuals (Yu et al., 2024). An extensive multicenter study, also based on REST-meta-MDD, found that the mean value of nodal efficiency in the thalamus was significantly abnormal in patients with MDD compared to healthy individuals (Zhou et al., 2024). Impaired top-down emotion regulation due to thalamic neuroarchitectural changes may be one of the etiological factors for patients with MDD (Webb, Weber, Mundy, & Killgore, 2014). In addition, several studies have found that the thalamus is associated with anxiety. For example, Langhammer et al. found that connectivity between the thalamus and insula was significantly increased in patients with anxiety disorders (Langhammer et al., 2024), suggesting that the thalamus plays a modulatory role in anxiety to some extent. This mechanism may be related to major projection sites in the medial nucleus of the thalamus, which include the prefrontal cortex, hippocampus, and amygdala regions that are closely associated with emotions such as anxiety and depression (Boelens Keun et al., 2021; W. Liu et al., 2025). This relationship could explain the significant differences in anxiety levels between the two FEDN MDD subtypes that we found based on structural covariation edges within the thalamus.

In addition, our present study found that subtype 1, which has significantly higher anxiety levels, exhibited lower GMV in the frontal lobe, parietal lobe, and thalamus. The frontal lobe is a core region of the human brain characterized by its large size and complex gyrus structure, which plays a crucial role in cognitive control and emotion regulation in patients with MDD (Catani, 2019; Cheng et al., 2016; C. Li et al., 2025). In patients with MDD, frontal and limbic circuits are thought to be intricately linked to emotion regulation. These circuits integrate bottom-up and top-down mechanisms associated with MDD and are critical to understanding the pathophysiology of this condition (Aleksandrova, Wang, & Phillips, 2019; Lai, 2021). The importance of this key brain structure has been emphasized by both machine learning studies and basic experimental research (Aleksandrova et al., 2019; Gao, Calhoun, & Sui, 2018). Studies have shown that the frontal and parietal lobes are key components of the Theory of Mind sub-network, influencing patients’ social cognitive and social perceptual abilities (Lai, 2021; Wang, Wang, Chen, Zhu, & Wang, 2008). This may represent an important aspect of the pathophysiological basis of MDD. The two FEDN MDD subtypes identified in this study showed differences in anxiety symptoms and structural differences in the aforementioned brain regions. Furthermore, correlation analyses indicated a potential relationship between these structural changes and anxiety symptoms. However, the link between the anxiety-depression subtype (which has previously shown structural changes) and the subtypes defined in the present study has not been thoroughly investigated (Juan et al., 2024; Qiao et al., 2020). Future research needs to further explore the potential link between the newly defined subtypes in this study and widely recognized MDD subtypes such as anxious depression.

Several limitations should be acknowledged in this study. First, our assessment was limited to anxiety and depressive symptoms in MDD patients. Due to the limited availability of detailed scale data from some research institutions in the REST-meta-MDD consortium, we were unable to analyze individual dimensions in the HAMD-17 and HAMA-14 in more detail. Future studies should incorporate more diverse assessment tools to allow for more comprehensive and nuanced analyses of MDD patients. Second, although we explored the neuroimaging and clinical characteristics of the two subtypes identified in this study, our analyses did not extend to the genetic level, which distinguishes our work from similar studies in the field. This is an important area for future investigation within the framework of the FEDN MDD study.

## Conclusions

In this study, we conducted cluster analysis based on individual-level characteristics, utilizing each patient’s unique IDSCN to identify patients with FEDN MDD into two stable subtypes. These subtypes exhibited distinctive neuroanatomical features and clinical symptom profiles. Notably, this subtype differentiation has often been overlooked in previous population-based analyses. Our multicenter, large-scale study not only revealed significant neuroimaging heterogeneity in FEDN MDD patients at the individual level but also provided valuable insights for advancing personalized therapeutic strategies and precision medicine approaches in MDD treatment.

## Data Availability

The data used in this study were obtained from the official REST-meta-MDD website (https://www.scidb.cn/en/detail?dataSetId=cbeb3c7124bf47a6af7b3236a3aaf3a8).
